# Squeezing the most from every crystal: the fine details of data collection

**DOI:** 10.1107/S0907444913013280

**Published:** 2013-06-18

**Authors:** Tobias Krojer, Ashley C. W. Pike, Frank von Delft

**Affiliations:** a Structural Genomics Consortium, Oxford University, Roosevelt Drive, Oxford OX3 7DQ, England; b Diamond Light Source, Harwell Science and Innovation Campus, Didcot OX11 0DE, England

**Keywords:** data collection, data-collection strategy, structural genomics

## Abstract

Modern synchrotron beamlines offer instrumentation of unprecedented quality, which in turn encourages increasingly marginal experiments, and for these, as much as ever, the ultimate success of data collection depends on the experience, but especially the care, of the experimenter. A representative set of difficult cases has been encountered at the Structural Genomics Consortium, a worldwide structural genomics initiative of which the Oxford site currently deposits three novel human structures per month. Achieving this target relies heavily on frequent visits to the Diamond Light Source, and the variety of crystal systems still demand customized data collection, diligent checks and careful planning of each experiment. Here, an overview is presented of the techniques and procedures that have been refined over the years and that are considered synchrotron best practice.

## Abstract 2

This article gives an overview of techniques and procedures for efficient data collection at synchrotron sources.

## 1. Introduction

Recent decades have seen an astonishing improvement in terms of synchrotron performance, beamline instrumentation and data-processing software. Protein crystallography is now a mainstream research technique and the rate of structure deposition in the Protein Data Bank keeps increasing (Abad-Zapatero, 2012 ▶). Nevertheless, despite much automation, data collection remains a nontrivial experiment that requires diligent planning and careful checks, while the brightness and brilliance of third-generation synchrotrons pose a whole additional set of challenges (Chavas *et al.*, 2012 ▶; Dauter *et al.*, 2010 ▶; Fodje *et al.*, 2012 ▶; Gabadinho *et al.*, 2010 ▶; González *et al.*, 2008 ▶; McCarthy *et al.*, 2009 ▶). The availability of ever smaller and brighter beams has boosted the field on the one hand, but it has also increased the requirement of the experimenter to make the correct choices at the beamline. The experiment potentially remains tricky and still requires experience and quick, accurate decisions.

The validity of this truism can be well observed in large research laboratories dedicated to structure solution, such as the Structural Genomics Consortium (SGC). The Oxford site of this international initiative currently deposits three (historically six) novel human protein crystal structures per month, and its productivity depends crucially on frequent visits to the state-of-the-art beamlines at the Diamond Light Source and, before 2009, to the Swiss Light Source. In terms of operational efficiency, while the cornerstone of the whole gene-to-structure process is to work in parallel, we have consistently found that if diffracting crystals have been identified then the most effective route to reducing additional biochemical effort is to approach data collection with extreme diligence in order to obtain the best data possible.

Thus, the output of a structural genomics project is not sustained by indiscriminately collecting vast amounts of data and hoping that their sheer number will eventually lead to the desired number of structures, contrary to the popular sense invoked by the term ‘high-throughput’ that is often used in the same context. As is frequently pointed out, the collection of diffraction data is the last real experiment and errors made at this stage may make structure solution unnecessarily difficult or even impossible. Indeed, by focusing on collecting few but high-quality data sets, we found that not only is the data analysis itself accelerated, but also the average amount of wet laboratory work required per project is greatly reduced; the time consumed by attempting to evaluate large quantities of mediocre data can never be overestimated.

Here, we provide an overview of the methods and routines that we have applied and refined over eight years of data collection at both the Diamond Light Source and the Swiss Light Source. This topic has been discussed extensively in the literature (Dauter, 1999 ▶, 2010 ▶; Evans, Axford, Waterman *et al.*, 2011 ▶; Pflugrath, 2004 ▶; Rupp, 2010 ▶; Mueller *et al.*, 2007 ▶) and much of this will be obvious to experienced synchrotron users; certainly not everything is crucial to every situation, but we have consistently found that shortcuts taken by impatient operators have come back to haunt them and their projects.

## 2. Homework

By far the most reliable route to good data is to have a good crystal, so effort spent in finding one before coming to the synchrotron generally pays off. Crystal morphology is not a predictor of diffraction quality, and one should therefore always explore all crystals grown in different crystallization conditions. Moreover, there are many ways to generate variant crystals, *e.g.* crystallization in the presence of different ligands (Vedadi *et al.*, 2006 ▶), crystallization of different constructs (Gileadi *et al.*, 2007 ▶), seeding (D’Arcy *et al.*, 2007 ▶), surface mutagenesis (Derewenda, 2010 ▶), lysine methylation (Walter *et al.*, 2006 ▶), diversified screening approaches such as the ‘silver bullets’ screen (McPherson & Cudney, 2006 ▶) and *in situ* proteolysis (Wernimont & Edwards, 2009 ▶). Post-crystallization procedures such as dehydration or annealing can in some cases lead to impressive improvements in diffraction quality (Heras & Martin, 2005 ▶; Sanchez-Weatherby *et al.*, 2009 ▶). Crystals should be mounted with great care and adhering to some simple rules will always be beneficial for the quality of a data set without any additional overheads (Pflugrath, 2004 ▶). A few points are worth emphasizing.

(i) The size of the loop should correspond to the size of the crystal, since the presence of excessive amounts of mother liquor around the crystal increases the background scatter and thus degrades the signal, especially in the high-resolution reflections (Garman, 1999 ▶); generally, the beam should traverse roughly equal amounts of crystalline and noncrystalline material. This guideline presupposes a very observant experimenter, since many crystals (especially thin plates and needles) suffer degraded diffraction when constrained to a drop that is too small and thus with too curved a surface. On the other hand, smaller crystals (<100 µm) appear to be more robust to such effects, possibly because their shortest dimensions are generally smaller than the thickness of the material of the mount, allowing them to sit inside the hole and remain unaffected by surface effects; happily, such crystals predominate in the smaller crystallization drops (150 nl) that are standard at the SGC, and the beamlines at Diamond have beams with sizes that are appropriate for such crystals. We have found that carefully organizing our loops in boxes labelled according to size has greatly reduced the number of tiny crystals that end up mounted in much larger loops and surrounded by a vast excess of solvent.
(ii) We mount most of our crystals in nylon loops (Hampton Research, California, USA) as they survive many tens of reuses, unlike commercial mounts made from other materials; however, thin plates or thin rods are best mounted in the more specialized mounts which are designed to match these crystal shapes exactly (MiTeGen, New York, USA).
(iii) While a standard (and often laboratory-specific) protocol for cryoprotection may be suitable for many crystals (in our hands, an excellent first choice is simply to add 25% ethylene glycol or 30% glycerol to the reservoir solution and to place an excess on the crystallization drop), it is often necessary to test different organic solvents or devise a more elaborate cryoprotection scheme (Pflugrath, 2004 ▶).
(iv) Hyperquenching during crystal cooling should be considered, especially if the crystals do not tolerate high concentrations of cryoprotectant (Warkentin *et al.*, 2006 ▶). It is extremely simple to set up and can be very effective in difficult cases; at the same time, experimenters may not be convinced of its necessity as a default approach.
(v) If diffraction from frozen crystals is unsatisfactory, one should assess their diffraction at room temperature in order to exclude problems during freezing; this is extremely convenient with the loop-based system sold by MiTeGen. Additionally, although it is in most cases impossible to collect a complete data set from a single unfrozen crystal at the synchrotron, today it is easily forgotten that multi-crystal data collection (Kendrew *et al.*, 1960 ▶) was how all structures were solved before the advent of cryo-techniques; happily, this approach is now experiencing a resurgence (Liu *et al.*, 2011 ▶, 2012 ▶; Ditzel *et al.*, 1998 ▶).
(vi) Where a long unit-cell axis requires a special crystal orientation (see, for example, §[Sec sec6.4]6.4), and if the axis orientation can be inferred from the crystal shape, one can attempt to orient the crystal in the mount in as favourable an orientation as possible relative to the pin. For instance, hexagonal cells with a very long c* axis frequently lead to crystals that grow as flat hexagonal plates, so that the default orientation in a flat loop places the c* axis unfavourably, namely perpendicular to the default axis of rotation; however, this can be counteracted with some dedication.

**Figure 1:**
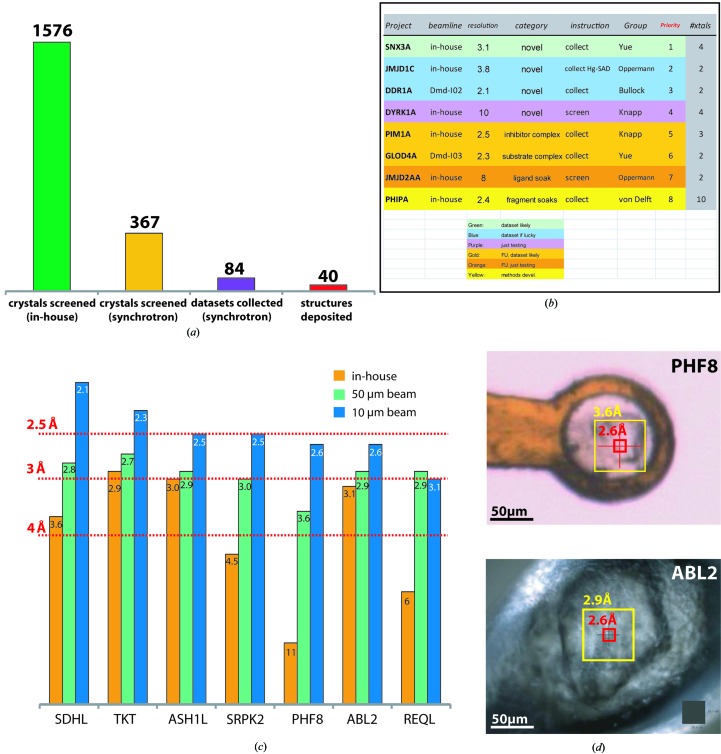
Efficiencies achieved by careful pre-visit preparation. (*a*) The distribution of diffraction experiments performed by the SGC throughout 2011 illustrates the value of testing crystals in-house: three quarters of crystals could be discarded as inferior, allowing beamtime to be dedicated to the collection of high-quality data sets, so that half led to deposited structures. (*b*) A well structured list of priorities such as this example from our laboratory, prepared and distributed in advance of the visit, greatly speeds up experiments by enabling decisions to be made rapidly. (*c*) A comparison of the best resolutions observed for a series of projects with various beams (see legend) illustrates that diffraction can generally be reliably identified from less intense beams and that improvements may actually be quite modest. (*d*) The very different improvements in resolution observed for crystals from two different projects using a trimmed and microfocused beam (*c*) can be rationalized by the ratio of the crystal size to the respective beam sizes: if the crystal is already much larger than the beam (ABL2) an even smaller beam will not help much.

Modern synchrotrons, in combination with sample-changing robotics, allow the rapid screening of many crystals at the beamline, but at SGC the policy remains that all crystals have to be first characterized for diffraction quality (although not necessarily space group) on our in-house rotating-anode generator to allow them to be prioritized for the synchrotron; unscreened crystals generally receive low priority (Figs. 1 ▶ *a* and 1 ▶ *b*). We have found this to be crucial as it allows us to focus our efforts and attention during the limited period of a beamtime on the actual collection of data. State-of-the-art rotating-anode generators often allow a realistic assessment of the quality of protein crystals: the diffraction observed for crystals larger than 100 µm on the Rigaku FR-E generator at the SGC (300 µm beam size) is typically between 0.5 and 2 Å poorer in resolution than that observed at the synchrotron, and spectacular improvements are only observed for crystals smaller than 50 µm (Figs. 1 ▶ *c* and 1 ▶ *d*). Additionally, pre-screening helps to exclude salt crystals, to identify issues with freezing or to identify crystal problems early on. Samples are prioritized taking account of both project importance and in-house screening results, so generally it is clear what kind of data-collection approach will be required for each project and how much time it will require even before the synchrotron visit starts. This procedure ensures that the time at the synchrotron is used as efficiently as possible.

## 3. Mechanical alignment of the beam with the spindle axis

Modern beamlines are remarkable machines, and we have found beamline support and maintenance to be consistently excellent worldwide, not only at our usual ‘home’ synchrotrons. Nevertheless, ‘drift’ is an unavoidable phenomenon for any system where movements of 10 µm or less are important, and since these are the typical experimental dimensions at modern synchrotrons, both the beam and the rotation axis can easily drift from the intersection point marked on the screen. It is therefore crucial that the user understands the beamline and takes responsibility for being alert to whether the beam is in fact hitting the crystal at all times and knowing how to diagnose and correct this both before and during the experiment. Even for the Diamond beamlines, whose stability we are very familiar with, we set aside time at the start of each visit for a start-up routine to check some critical features together with the beamline scientists; if nothing else, this reminds us as users how to control the beamline and diagnose it once we are on our own during the rest of the visit.

On-axis viewing systems (Perrakis *et al.*, 1999 ▶) offer the extraordinary benefit that users are able to directly visualize the crystal-to-beam intersection. The beam position can easily be checked by mounting an X-ray sensitive screen to verify that the beam is actually at the position of the crystal, and many beamlines have motorized YAG screens that can be rapidly placed in the beam, even while the sample is on the goniometer. What should also be investigated is how different slit or aperture settings affect both the shape and the position of the beam; even if the viewing software draws outlines of the apparent beam, these will necessarily only be approximations of the actual beam profile. It is advisable to repeatedly review the position of the beam over the course of a visit, especially when working with small samples; when beams drift, this happens on the timescale of hours, and if the beam position is not regularly checked this can easily lead to false-negative results. It is also important to be familiar with how to correct for such drifts when they occur.

Equally important is to assess the sphere of confusion: the alignment of the rotation axis and its rotational accuracy. A large sphere of confusion will cause the sample to ‘wobble’ around the beam, and assessing it requires a well defined point mounted on the goniometer; typically, beamlines have sharp needles mounted on standard bases available and we have also found it useful to carry our own. On a well aligned rotation axis, the tip of the object (needle) should stay in the marked beam centre for an entire 360° oscillation, but mechanical limitations or a slight offset of the axis may prevent this. The latter is easily corrected by the beamline controls, but the former is a feature of the instrument and this rotation-dependent deviation may need to be taken into account when planning a particular diffraction experiment, especially for very small crystals and experiments that require a full 360° rotation (§[Sec sec4]4).

## 4. Alignment of sample to beam

The most fundamental aspect of the experiment is that the beam must properly intersect with the sample for the entire oscillation. This is not a problem when the crystals are much larger than the beam, but with small crystals and small beams it is easy to get wrong. When aligning visually, the main obstacle to aligning the sample is parallax owing to the shape of the liquid surrounding the crystal in the loop; the only reliable view of the crystal is perpendicular to the loop, and identifying this orientation should be the first step during the alignment of each sample. The quickest way to do so is to find the ‘side-on’ direction, since it is most readily distinguished; from there, a 90° oscillation brings up the ‘face-on’ view. Any alignment should be confirmed by viewing the loop from the other side, *i.e.* by applying a 180° rotation.

However, the side-on orientation presents a fundamental problem whenever the crystal does not extend between both surfaces of the liquid in the loop or when it is thinner than the thickness of the loop material itself. In this case, diffraction must be used to locate the crystal by scanning X-rays across a region of interest and measuring the diffraction strength (Aishima *et al.*, 2010 ▶; Hilgart *et al.*, 2011 ▶). Low doses are recommended for such scans in order to avoid unnecessary X-­ray damage, since only a small fraction of the photons required for an image in a properly measured data set are sufficient to identify the strongest diffraction. While automated analyses are helpful when available, we recommend visual inspection of the diffraction pattern, especially for low-dose exposures. This is easily performed by opening an image in a diffraction-image viewer, selecting an obvious Bragg spot, zooming in to view the actual pixel counts and then finding the image with the highest number of counts in the spot.

Grid scans also offer the opportunity to characterize diffraction throughout the crystal; in our experience, crystals very commonly diffract inhomogeneously, especially those that are poorly ordered and thus diffract to low resolution, regardless of their morphological appearance. Crystals of membrane proteins appear to be particularly prone to this phenomenon (Evans, Axford & Owen, 2011 ▶), and even when large crystals can be generated the use of the microfocus beam of I24 remains informative.

**Figure 2:**
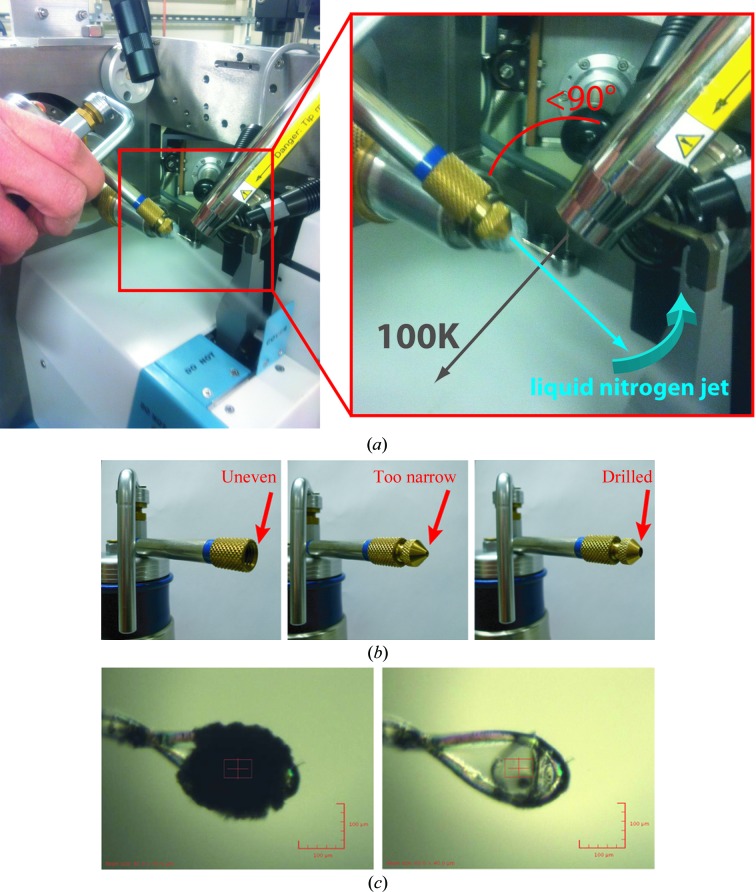
Removal of surface ice with a jet of liquid nitrogen from a BryMill cryosurgery device. The process is efficient and safe, provided that it is performed correctly. (*a*) In order not to blow away the cold air stream the jet should be at an acute angle to it, and it should not be pointed directly at the crystal but instead swept towards it across the cold air stream. There must be sufficient pressure so the jet does not splutter and large loops should be turned so their edge faces the jet, because the sample drop does not stick well to the loop. (*b*) A nozzle with a non-uniform edge will produce a divergent jet which may blow the cold stream away and lead to destruction of the sample; a quick remedy is to drill out one of the inserts supplied. (*c*) Image of the loop before and after deicing.

Visual alignment encounters two other common obstacles. (i) Loops that are significantly bent away from the rotation axis will present further parallax, and to view these reliably may require manual intervention to straighten the loop or else the use of kappa goniometry where available (see §[Sec sec6.4]6.4). (ii) Surface ice obscures the crystal, but also degrades the diffraction pattern with strong powder diffraction rings. We have been able to remove it routinely by blowing it away with a stream of liquid nitrogen using a Cry-Ac cryosurgery device (Brymill, Connecticut, USA; Fig. 2 ▶; James Holton, personal communication). Be aware, though, that the cold gas stream may damage the beamline equipment and the use of such a device should be discussed with the beamline scientists beforehand. Crystal ‘washers’, which flood the crystal with a stream of liquid nitrogen on demand, are also becoming more widespread at beamlines (Diamond Light Source, personal communication).

Use of very small crystals on beamlines with a sphere of confusion of similar magnitude to either the crystal or the beam requires special attention: either angular ranges with the largest offset need to be avoided or else several re-alignments may need to be performed in the course of a 360° data set either side of a particular oscillation range (we use scans of 90°). Care also has to be taken when setting up helical data collection (Evans, Axford, Waterman *et al.*, 2011 ▶; Flot *et al.*, 2010 ▶; see §[Sec sec6.5]6.5): for such scans on thin rods on a microfocus beamline the end points need to be determined with the utmost care and diffraction-based alignment (Flot *et al.*, 2010 ▶; Hilgart *et al.*, 2011 ▶; Song *et al.*, 2007 ▶) is strictly required, since even small parallax may be sufficient to offset the apparent position of the crystal.

Another source of misalignment is more prosaic, namely defective sample mounts, where either the pin is loose in the base or the loop itself is loose in the pin; certainly this is worth investigating before instigating a more wide-ranging diagnosis of potential beamline failures. Incidence of this problem can be avoided by diligent pin management in the home laboratory, but for the inevitable exceptions, if a particularly valuable sample is involved, creative application of Plasticine may temporarily alleviate the problem.

## 5. Documentation of experiments

The need for rigorous documentation of diffraction experiments is easily overlooked during preparation for synchrotrons, yet it is of the utmost importance, since when it is neglected inconsistent practices will lead over time to a set of widely divergent notes that are difficult to search and are usually significantly incomplete. We have found it invaluable to develop a single documentation convention that has to be followed by every group member. Mostly, this merely requires only a little thought from an experienced synchrotron user in the group, and if the conventions are properly disseminated people are perfectly willing to adopt them.

The most basic aspect concerns files and directories, which can be judiciously named to be self-documenting. The use of arbitrary names is common (‘test1’, ‘crystal3’, ‘thing’), but this practice should be strongly discouraged since such naming will at best be informative to one individual, but will mostly mean the experiment cannot be reconstructed by anybody, including the creator after a few weeks. At SGC Oxford we adopted a naming convention that maps onto that of the *CCP*4 program suite (Winn *et al.*, 2011 ▶): each harvested crystal is given a unique crystal ID from a given project (*e.g.* BBOX1-x023 for project BBOX1) and each data set is given a unique data-set ID (BBOX1-d005), where a data set comprises one or more passes collected consecutively in time. These are combined for image names: BBOX1-d001-x003, BBOX1-d002-x047 *etc*. All of the data files are stored in a uniformly organized file system, which it is advisable to keep separate from where processing occurs: this greatly facilitates the management of disc space, since it makes it easier to locate the far larger data directories for deletion (after backup). Such practice is not encouraged by the default behaviour of the common data-integration packages, yet is nevertheless easy to perform in all of them.

Rigorous use of database infrastructure, where available, provides even greater benefits. SGC Oxford, which comprises around 80 researchers from diverse backgrounds ranging from cell biology, (bio)chemistry, informatics, engineering to crystallography, has benefited enormously from a central database (BeeHive; Molsoft LLC, La Jolla, California, USA) and an electronic laboratory journal (Contur ELN; Contur Software AB, Sweden). Every step of a project from target selection to structure deposition can be rapidly reconstructed through the query engine, because this has been the only formal experimental record accepted in the laboratory from the start. The consistent utility that this infrastructure has provided, not least for real-time decision-making, prompts us to submit that such systems should be a strict requirement in any large operation, whether industry or academic efforts such as the SGC; even only a subset of functions would provide enormous benefit to smaller research groups. Freely available database systems are available, including a publicly hosted server of PIMS (Morris *et al.*, 2011 ▶), and many synchrotrons offer database support systems such as ISPyB, which record all details of a diffraction experiment (Delagenière *et al.*, 2011 ▶). However, the usefulness of such a system stands and falls with the willingness of the head of the laboratory to enforce both its use and the consistency of the corresponding naming conventions.

## 6. Data collection

Even when the crystals have been pre-screened in-house, it is very important to reassess at least several of the best crystals before investing the time to set up and execute data collection. Modern sample changers are extremely reliable and we cannot confirm anecdotal reports that dismounting crystals can lead to loss of diffraction: in more than 650 diffracting projects, crystals that diffracted before being returned to liquid nitrogen, invariably still diffracted just as well when replaced on the goniometer. Indeed, rather than introducing risks, a willingness to remove cryopreserved crystals from the gonio­meter once tested, allows synchrotron time to be used much more efficiently.

Another potential pitfall is presented by the revolutionary speed of modern detectors, especially pixel-array detectors, which makes it possible to collect many more data sets in the allotted beamtime than in the past. This capability is a powerful tool, but we also observe that, at least for unphased structures, meticulous planning of the diffraction experiment cannot be dispensed with and cannot be compensated for by ‘data-set roulette’ *i.e.* the collection of data as quickly as possible from as many crystals as possible in the hope that one is good enough. Moreover, data processing can become very time-consuming or even impossible when crystals are suboptimal, suffer from radiation damage or are poorly aligned, and ultimately no time is saved.

Finally, it is important to become closely familiar with the necessary software, experimental requirements and strategy options before arriving at the synchrotron, because once there there is always great time pressure, and the ability to generate and understand real-time diagnostics often makes the difference between success and failure. Historical data sets from other projects in the laboratory are an excellent resource for learning the software.

### 6.1. Initial characterization

Once a sample is properly aligned we record two diffraction images that are 90° apart, since frequently diffraction appears to be good in one crystal orientation but is problematic in the perpendicular direction. For this reason, careful visual inspection of diffraction is important using an image viewer such as *ADXV* (http://www.scripps.edu/~arvai/adxv.html) or *ALBULA* (https://www.dectris.com/software_albula.html) to identify diffraction pathologies such as split spots, multiple lattices, smeary spots, ice rings or anisotropic diffraction.

**Figure 3:**
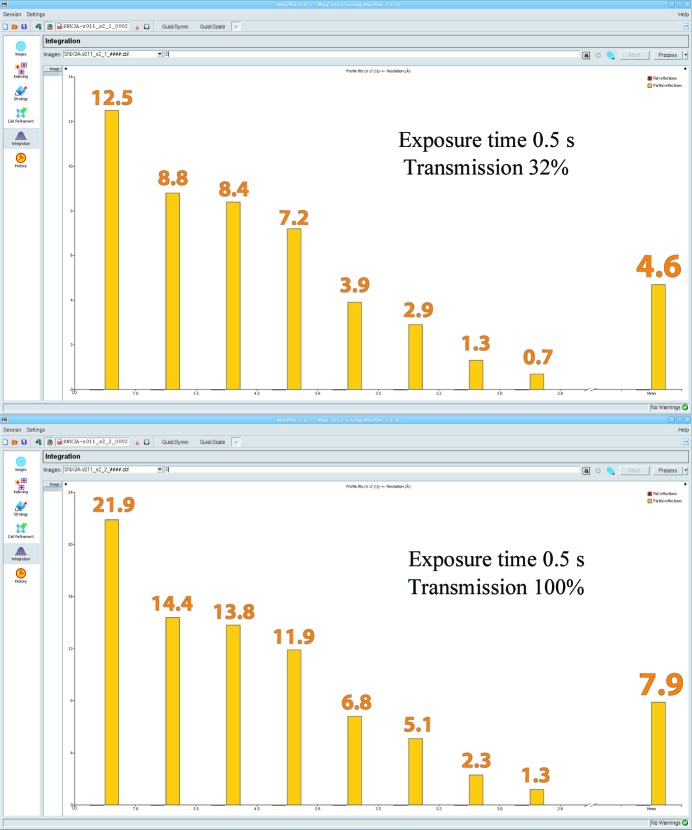
Evaluation of diffraction resolution using single-image integration in *iMosflm*: the histogram shows *I*/σ(*I*) for each resolution bin, which increases correspondingly when the flux is increased (compare the upper and lower panels). In the absence of decay, the resolution where *I*/σ(*I*) ≃ 1 on a single image will generally yield *I*/σ(*I*) ≃ 2 when the whole data set is merged.

Although beamline staff can advise on typical exposure times and transmission settings for their beamline, this will not necessarily allow adequate characterization of a given crystal and, especially when diffraction is weak, higher fluxes should be explored to see whether reflections start to appear at higher resolution or whether there is just an increase in background intensity. For a semi-quantitive assessment, the program *iMosflm* (Battye *et al.*, 2011 ▶) provides the invaluable facility of a graphical view of *I*/σ(*I*) per resolution bin when integrating even only a single image (Fig. 3 ▶), provided that the program succeeds in indexing the diffraction. This exercise has the added advantage of allowing a visual comparison between the predicted and observed diffraction, which is easy to overlook when relying on nongraphical (scripted) integration.

If attempts at indexing fail, it may be better to search for a better crystal; on the other hand, if there are no better crystals it is certainly worth proceeding with data collection, since frequently data processing does then succeed when all images of the data set are available.

### 6.2. Beam size and shape

An ideal diffraction experiment would have the beam illuminate only the crystal and no more, since all unnecessarily illuminated material will contribute background scattering and degrade the measured signal. If the beamline provides the option to change the beam size easily, this should be explored, including a semi-quantitive assessment of the resolution limits detailed in §[Sec sec6.1]6.1. In particular, one needs to be aware how the beam size is related to the beam flux, since beamlines achieve changes in beam size either by cutting the beam, which reduces the flux, or by refocusing, which preserves the flux; the exposure time or transmission may need to be adjusted to compensate accordingly, sometimes by several orders of magnitude.

Appropriate tailoring of beam size and shape can also be critical for obtaining good data from inhomogeneous crystals once a volume of good diffraction has been identified (§[Sec sec4]4). Additionally, one can consider adjusting the beam size as the crystal rotates and presents a changing profile to the beam, and the exposure times need to be adjusted too; this has not been our standard practice, although in extreme cases it may significantly improve data quality and thus be worth the effort of setting up.

### 6.3. Geometric strategy: oscillation range

Although a 180° oscillation will always yield a set of images in which all accessible indices have appeared at least once, and although this can be achieved within seconds on bright beamlines with fast detectors (Owen *et al.*, 2012 ▶), it is critical to realise that the crystal may have deteriorated well before the experiment is over. The consistent undermining factor is radiation-induced decay and the fact that the lifetime of the crystal is in general unknown beforehand. Hence, a sensible data-collection strategy is to use the minimal angular range required to collect a complete data set for the probable Laue group, as this maximizes the likelihood of measuring all of the required reflections before radiation damage dominates. If anomalous signal is to be measured, it is always beneficial to maximize the observational multiplicity by collecting as large an oscillation range as practically feasible, and even multiple revolutions at different crystal offsets (Debreczeni *et al.*, 2003 ▶; §[Sec sec6.4]6.4), adjusting the dosage strategy accordingly (§[Sec sec6.5]6.5).

When targeting high resolution it is the counting statistics of the weak reflections that is limiting (Holton, 2009 ▶), and it suffices to ensure that all reflections are measured. However, one should always collect a larger angular range than that prescribed by the geometric strategy in case the crystal does survive the minimal range, because increased multiplicity will yield improved data accuracy and signal to noise (Garman, 1999 ▶). If the later images of the data set do end up overly compromised by radiation damage, they can be removed during the final data processing.

All data-processing packages can calculate such strategies, and it is recommended to work with the package that one is most familiar with. A more sophisticated strategy is provided by the program *BEST* (Bourenkov & Popov, 2010 ▶), which is most typically available *via* the automated *EDNA* software (Incardona *et al.*, 2009 ▶); it not only calculates the geometric strategy but also provides recommendations for exposure time and predicts the outcome of the experiment. 

In some cases, especially for crystals with low symmetry, the crystal orientation means that no single oscillation range will yield complete data. In these instances, it is necessary to change the crystal orientation (§[Sec sec6.4]6.4) and to collect data for a second oscillation. Strategy programs do allow this to be calculated, but making use of the information requires dedication and patience, unless the beamline is set up appropriately, *e.g.* with the *STAC* software (Brockhauser *et al.*, 2011 ▶); also, the crystal must be able to survive additional irradiation.

### 6.4. Overlap strategy: oscillation angle

Another important consideration is the oscillation angle per image, in order to avoid spatial overlap of diffraction spots on the detector. The availability of pixel area detectors makes it feasible to use fine slicing (Pflugrath, 1999 ▶) by default, and apart from the potential gains in data quality and signal to noise (Mueller *et al.*, 2012 ▶), this also greatly reduces the risk of spot overlap.

However, even fine slicing will not help when a sufficiently long unit-cell axis is oriented perpendicular to the beam: in such cases the geometry ensures that there will be spatial overlap even for still images, with no guarantee that any algorithm can deconvolute the overlapped intensities reliably. This particular problem is easy to spot with an interactive program such as *iMosflm*, as it displays the percentage of overlaps for a given oscillation angle as long as the mosaicity has been properly estimated.

**Figure 4:**
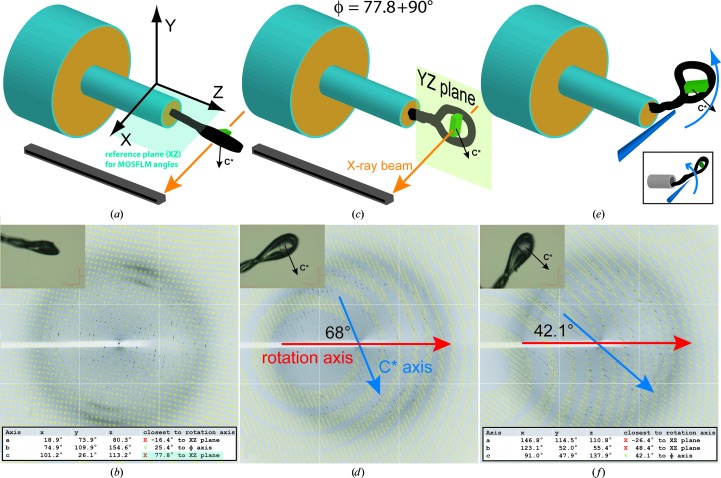
Crystal reorientation to avoid spot overlap owing to a long unit-cell axis oriented close to the beam: after the long axis (in this example c*, oriented in the plane of the loop) has been rotated into the plane perpendicular to the beam, the cell axis can be brought more parallel to the spindle axis by bending the loop in that plane. (*a*) Schematic representation of the goniometer showing the crystal in an arbitrary orientation, along with the laboratory reference frame conventions used by *iMosflm*. (*b*) Diffraction pattern and crystal view (upper inset) from the arbitrary crystal orientation, showing the close spacing of predictions characteristic of a long axis oriented close to the beam; strategy analysis in *iMosflm* indicates that data collection will be compromised by overlaps (not shown); it also provides (lower inset) the rotation required to bring the long axis into the *iMosflm* reference plane *XZ* (blue highlight). (*c*) Schematic showing the crystal rotated to place the long axis into the desired *YZ* plane: since it is perpendicular to the *iMosflm* reference plane, 90° is added to the angle indicated in (*b*). (*d*) Diffraction pattern of the rotated crystal, with the orientation of the long c* axis now clearly identifiable at 68° from the spindle axis; this angle needs to be reduced. (*e*) Schematic of how to reorient the crystal manually, using a sharp point to bend the loop in the required direction by pushing at where it is attached to the metal pin (care should be taken not to breathe away the cold stream!). Bending works best when pushing the nylon against the edge of the pin; the insert shows the less effective direction when bending towards the centre of the pin. Of course, a kappa goniometer, if present, allows the crystal to be tilted far more conveniently and accurately. (*f*) Diffraction pattern of the reoriented crystal, showing the long c* axis now significantly closer to the spindle axis, sufficient in this case to avoid spot overlap in the whole data set.

This long-axis problem can only be circumvented by re­orienting the crystal: this is straightforward with a goniometer that supports κ offsets (*e.g.* a kappa motor), although many modern beamlines do not have this facility. Alternatively, if one has previous knowledge of how the crystal morphology is related to the unit-cell axes, one can try to mount the crystal in a favourable orientation (§[Sec sec2]2); some manufacturers (*e.g.* Hampton Research, California, USA) sell special bendable loops for this purpose. However, in the absence of all of this (and with a bit of practice) it is very feasible to bend nylon loops while they are mounted on the goniometer (Fig. 4 ▶; Dauter, 1999 ▶). This naturally cannot achieve any angular accuracy, yet the procedure has proved itself to be adequate for a significant number of projects in which reorientation was crucial to success but where kappa goniometry was not available. Non-nylon sample mounts are much less amenable to this procedure.

### 6.5. Dosage strategy: transmission and exposure time

At third-generation synchrotrons, where photon flux is no longer limiting, the specific question of how much X-ray exposure to apply per image or unit of oscillation is almost entirely one of how much radiation damage will be tolerated by the crystal, or indeed the atoms with anomalous scattering when measuring anomalous signal.

General radiation damage is characterized by the fading of high-resolution reflections over the course of data collection (Owen *et al.*, 2006 ▶), and the highest resolution reflections observed on an initial image, usually taken with high flux, seldom survive for the duration of the data set. Thus, the initially estimated resolution is rarely achieved for the whole data set, particularly for smaller crystals (Holton, 2009 ▶). Additional technical but also scientific problems arise from site-specific radiation damage of certain chemical groups in the crystal, and such effects may start much earlier and may restrict the usefulness of the final structure (Weik *et al.*, 2000 ▶; Ravelli & McSweeney, 2000 ▶) or else the anomalous signal. There is now extensive literature on this topic (Garman & Weik, 2013 ▶), including software that generates estimates of the total dose that the crystal will tolerate and therefore how to divide it up in the oscillation range (Paithankar & Garman, 2010 ▶; Bourenkov & Popov, 2010 ▶; see §[Sec sec6.3]6.3). However, such estimates are fully reliable only when the whole experiment is very carefully parameterized, which in general is not realistic (Krojer & von Delft, 2011 ▶). We therefore resort to an empirical approach, collecting an exploratory data set typically either from one end of the crystal or from a similar crystal of slightly less good quality and judging the radiation tolerance of the crystal from the data-processing statistics.

A rigorous implementation of this approach exists at the beamlines of the ESRF synchrotron as an automated option in the *MxCube* software (Brockhauser *et al.*, 2012 ▶; Leal *et al.*, 2011 ▶). If such a proper parameterization is not available, a useful estimate of the lifetime can nevertheless be gained by considering a few statistics and after how many images (or seconds) they reach a certain threshold.

(i) *AIMLESS* (Evans & Murshudov, 2013 ▶) prints out the maximum resolution bin for which *I*/σ(*I*) > 1 and, conservatively, the loss of resolution should remain below 0.2 Å if signal is to be present at the highest resolution after data from all images are averaged.
(ii) The scaling *B* factor directly models how resolution changes within the data set (*e.g.* relative to the first image), and its progression has been shown to relate directly to the absorbed dose (Kmetko *et al.*, 2006 ▶) at a somewhat invariant rate of ∼1 MGy Å^−2^ (Bourenkov & Popov, 2010 ▶). Since total loss of diffraction appears to occur between 20 and 30 MGy (Owen *et al.*, 2006 ▶), a conservative limit for practical purposes would be 10 MGy or lower, and much lower when anomalous signal is to be measured, since absorption and thus site-specific damage is greater for atoms that are centres of anomalous scattering. The scaling *B* factor is used, and therefore output by the programs *AIMLESS*, *SCALA* (Evans, 2006 ▶) and *HKL*-2000 (Otwinowski & Minor, 1997 ▶); a more complete description is given elsewhere (Krojer & von Delft, 2011 ▶).
(iii) The most important caveat to these estimates is that they do not account for diffraction anisotropy or crystal misalignment, both of which cause indicators of resolution to vary by rotation regardless of radiation damage; the scaling *B* factor is also very unreliable when scattering is weaker than ∼2.8 Å. Instead, collecting data for complete spindle revolutions (which is very realistic with detectors allowing shutterless data collection) allows both these effects to be factored out, since diffraction strength should be equivalent every 180° and 360°.
(iv) Simple spot finding on each image is crude but fast to calculate, as it does not depend on data integration and scaling. At Diamond, this is automatically generated for all images using *DISTL* (Zhang *et al.*, 2006 ▶) and prominently displayed at the beamline; we use a loss of ∼30% of spots as a cutoff if the metrics above could not be generated, although there is no theoretical basis for this value as it is a metric of last resort.

The observed lifetime must of course be corrected for the transmission setting used for the data set. Estimates of radiation tolerance have proven to be approximately transferable between beamlines for similarly shaped crystals of the same crystal system, if nominal beamline flux and the size of the beam are taken into account.

One route to avoid gauging radiation tolerance *a priori* is ‘dose slicing’, whereby the same oscillation range is re­measured repeatedly at low dose, with each pass receiving a fraction of the total tolerated dose. By integrating each pass individually, damage can be assessed with statistics from all reflections and only earlier passes are merged. Theoretically, this is no different to a single high-dose pass, and is also not much slower with modern zero-noise continuous-readout detectors. This approach is standard for systematic studies of radiation damage (Kmetko *et al.*, 2006 ▶; Owen *et al.*, 2006 ▶), but limitations remain for routine practical use, including the ability of integration programs to process images with very few photons, much increased disk-space requirements and the lack of well established cutoff criteria.

For crystals that are significantly longer than the beam is wide, many beamlines now allow helical data collection, whereby during rotation the crystal is translated through the beam along the rotation axis (Evans, Axford, Waterman *et al.*, 2011 ▶; Zeldin *et al.*, 2013 ▶). This increases the effective total crystal volume, allowing a higher total dose to be used for the data set; however, it does require the crystal to diffract uniformly (see §[Sec sec4]4). An estimate of radiation tolerance is still very useful, as it allows one to calculate how slowly the crystal can be translated, which in turn maximizes the yield of diffracted photons from a given crystal volume.

For CCD detectors, which have a lower dynamic range than hybrid pixel detectors, one has to be alert to the possibility of overexposure, since this will lead to individual pixels being overloaded. Consequently, the intensity of the affected reflections cannot be reliably established, leading either to wrong measurements or to otherwise incomplete data in the lowest resolution shells, depending on whether one instructs the integration program to reconstruct the intensity or to reject the reflections as ‘overloads’; both outcomes present significant problems for structure solution (Dauter, 2010 ▶). In such cases the approach should be first to collect a quick low-dose low-resolution data set that ensures that all of the reflections can be integrated reliably, followed by a high-dose pass for high-resolution reflections. For the latter, useful rules of thumb can be generated (Holton & Frankel, 2010 ▶), such as limiting exposures on ADSC Q315r detectors so that background levels do not exceed 100 counts (see equation 18 of Holton & Frankel, 2010 ▶). (Even pixel-array detectors can show a ‘temporal overload’ effect if diffracted photons ‘pile up’ by arriving too close in time, but this occurs only for beams consisting of extremely high-flux pulses, such as X-ray free-electron lasers, and we are not aware of MX beamlines where this is of practical relevance.)

For experiments where a full data set can be collected in a very few seconds, such as very strongly diffracting or low-mosaicity crystals on sufficiently intense beamlines, one should be aware of beam flicker, a high-frequency fluctuation of beam intensity which has many origins. A quick experiment requires the spindle to rotate so fast that each spot is in diffraction condition briefly enough to fall only within an intensity peak or trough of the beam, which introduces potentially significant systematic errors between related reflections. One relevant scenario is when collecting the ‘low-resolution’ pass and, for the sake of saving time, the crystal rotation speed is increased instead of reducing the beam transmission. Beam flicker is a complex property of any beamline and very difficult to characterize, let alone link to diffraction quality; it is easily avoided by maintaining reasonable oscillation speeds (>0.5 s deg^−1^ at Diamond beamlines) and attenuating the beam instead.

### 6.6. Crystal-to-detector distance

The detector should on the one hand be placed as far as possible from the crystal to dilute the background scattering of the direct beam by air, the sample mount and indeed the crystal itself, but close enough to record all possible reflections (Dauter, 2010 ▶). We usually take the best image from the initial characterization (see §[Sec sec6.1]6.1) and integrate it with *MOSFLM*. As a rule of thumb, we set the resolution limit to where the signal-to-noise ratio drops below 1 in this initial and somewhat crude assessment. Recently, it has been suggested (Karplus & Diederichs, 2012 ▶) that somewhat higher resolution data should be allowed than has been traditionally recommended; indeed, this probably matches common practice anyway (and certainly ours), since most experimenters tend to hope for better resolution than will probably be achieved; what is new is the recommendation to include such measurements in processing and refinement. Nevertheless, the distance should remain somewhat reasonable, *e.g.* not setting it for 2 Å resolution data when no spots are evident beyond 3 Å resolution.

## 7. Data processing and diagnostics

Given how many things can go wrong, it is absolutely crucial to be aware of whether the data being collected are of useful quality or not. In the best case, poor data allow an accurate diagnosis (*e.g.* misaligned beam, decaying crystal), allowing one to rerun the experiment and obtain better data; in the worst case one can recognize the futility of the project and proceed to the next one.

It is thus uncontestably best practice to attempt data processing in as close to real time as possible. Many synchrotrons offer not only excellent computing facilities but also automated data processing; the facilities at the Diamond Light Source have become indispensable for much of our work, particularly since the installation of continuous-readout PILATUS detectors (Winter & McAuley, 2011 ▶). However, the onus still remains on the user to actually look at the output, know how to interpret it, attempt to correlate it to the actual experiment and attempt data processing manually when automated methods fail.

## 8. Summary

This article emphasizes data collection from novel poorly characterized crystal systems. Of course, many shortcuts make sense when a system is well known, *e.g.* multiple protein–ligand complexes (often called ‘molecular substitution’; Wasserman *et al.*, 2012 ▶). However, for difficult projects we have found that one cannot be careful enough. Regardless of how easy it seems on modern facilities, X-ray data collection is actually a complicated experiment, and given how tiny the signal actually is, it is astonishing that we are able to measure what we do. The fact that this can nowadays be easily forgotten is a testament to the huge developments over recent decades.

Nevertheless, crystals that need special care will appear with great regularity; for these, an intimate knowledge of the experiment will remain crucial for many years to come.
